# Diet-driven microbiome changes and physical activity in cancer patients

**DOI:** 10.3389/fnut.2023.1285516

**Published:** 2023-11-24

**Authors:** Sona Ciernikova, Aneta Sevcikova, Viola Stevurkova, Michal Mego

**Affiliations:** ^1^Department of Genetics, Cancer Research Institute, Biomedical Research Center of Slovak Academy of Sciences, Bratislava, Slovakia; ^2^2nd Department of Oncology, Faculty of Medicine, Bratislava and National Cancer Institute, Comenius University, Bratislava, Slovakia

**Keywords:** gut microbiome, cancer, dietary interventions, treatment response, physical activity

## Abstract

Exploring the role of the gut microbiome in oncology is gaining more attention, mainly due to its ability to shape the immune system in cancer patients. A well-balanced microbial composition forms a symbiotic relationship with the host organism. Mounting evidence supports the potential of modifiable lifestyle factors, such as diet and physical activity, in restoring intestinal dysbiosis related to cancer development and treatment. In this Minireview, we describe the host-microbiome interplay following different dietary patterns, including a high-fat diet, fiber-rich diet, diet rich in rice and beans, Mediterranean diet, ketogenic diet, and physical activity in preclinical findings and clinical settings. According to the results, nutrition is a critical factor influencing the composition of gut microbial communities. Therefore, knowledge about the patient’s nutritional status in pre-treatment and treatment becomes crucial for further management. A combination of individualized dietary habits and professional training plans might help to maintain gut homeostasis, potentially improving the response to anti-cancer therapy and the quality of life in cancer survivors. However, a deep understanding of underlying mechanisms and large clinical trials are needed to uncover clinically relevant correlations for personalized treatment approaches leading to better outcomes for cancer patients.

## 1 Introduction

Tumor biology and the stage of the disease represent the key factors influencing the treatment response and outcomes for cancer patients (1). However, recent studies also highlight the critical impact of the gut microbiome, as well as microbes inhabiting tissues or organs, including mucosal surfaces of the body and tumor microenvironment, on the treatment efficacy (2–5). In 2022, polymorphic microbiomes were added to the newest version of “*Hallmarks of cancer*,” comprising common characteristics of tumors. As stated, microbiomes represent an enabling characteristic affecting other cancer hallmarks and modulating tumor phenotype (6).

The gut microbiome represents a complex ecosystem consisting of collective microbiota residing in the human gastrointestinal tract (GIT) with all the genetic material, metabolic functions, and interactions with the environment (7, 8). Dominant microbial phyla in the human intestinal tract are Firmicutes, Bacteroidetes, Proteobacteria, Fusobacteria, Actinobacteria, and Verrucomicrobia (9, 10). The Firmicutes/Bacteroidetes ratio might reflect potential microbiome-associated diseases (11). The production of microbiota-derived metabolites, including mainly short-chain fatty acids (SCFA), represents one of the key elements in host-microbiota crosstalk (12). This process is dependent on the concentration of fiber and other complex carbohydrates but also on intestinal composition and transit time.

**Figure 1:**
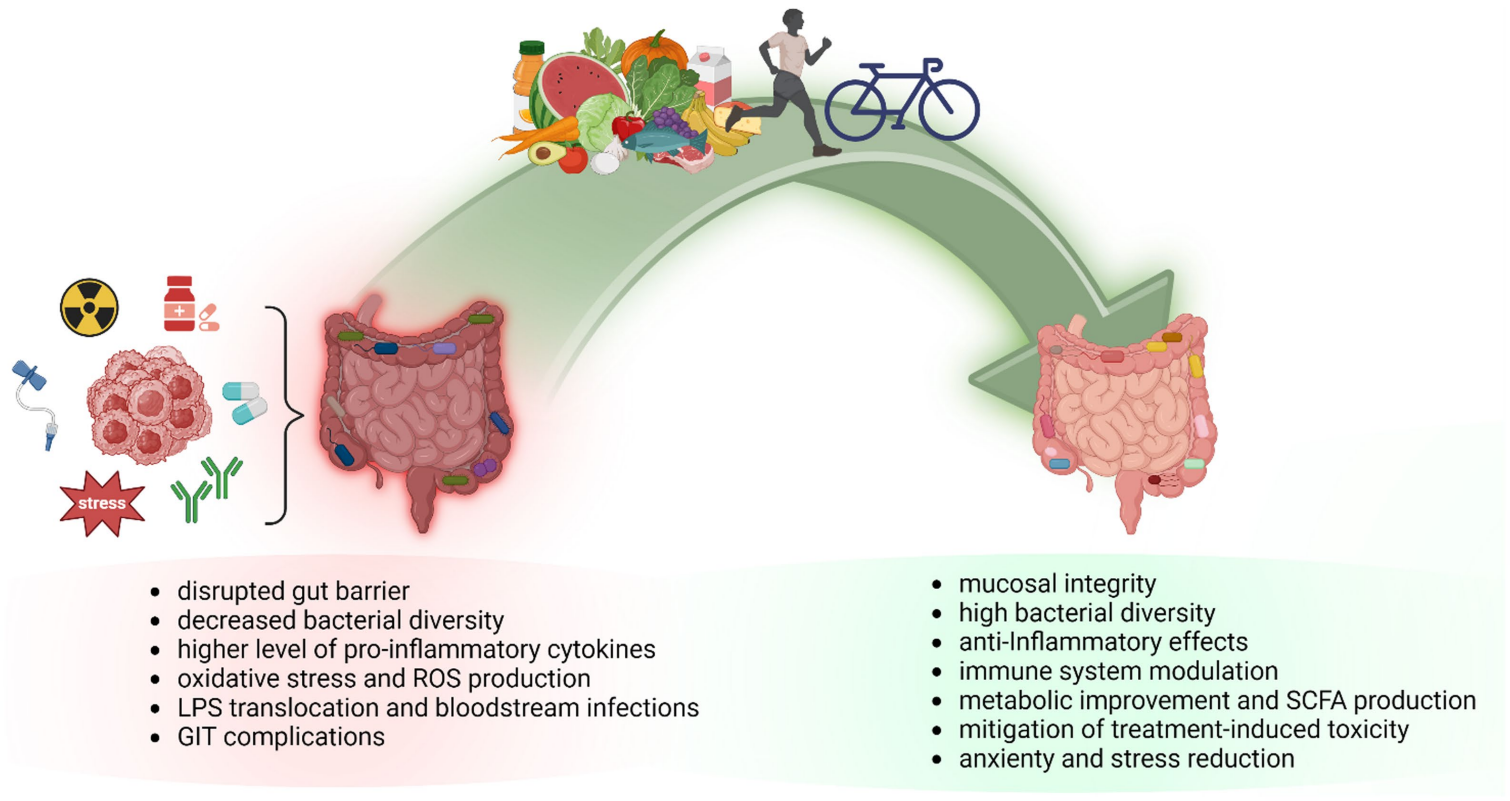
The impact of dietary intervention and exercise on gut microbiome composition in cancer patients. Gut dysbiosis is implicated in cancer development, and progression and negatively affects anti-cancer treatment resulting in worse patient prognosis. A well-balanced diet and regular exercise can modify the composition of the gut microbiome and elevate the levels of favorable microbes and microbiota-derived metabolites, leading to improved gut mucus layer integrity and decreased intestinal inflammation. Diet-microbiome interactions are involved in cancer prevention and development but also in response to anti-cancer treatment via gut-related anti-inflammatory and antioxidant activity. Physical activity guidelines aim to improve health conditions and quality of life in obese, overweight, or inactive cancer patients. As documented, exercise during cancer treatment might reduce mortality risk, and physical activity in cancer survivors decreases the risk of cancer recurrence. GIT, gastrointestinal tract; LPS, lipopolysaccharide; ROS, reactive oxygen species; SCFA, short-chain fatty acids.

Besides oncologic disease, anti-cancer and supportive treatment modalities and antibiotic prophylaxis contribute to major shifts in gut microbiome composition. Data emphasize the relevance of microbiota modulation in cancer survivors via lifestyle-related changes (Figure 1) (13–15).

Nutrition critically shapes the human gut microbiome composition (16, 17). Wastyk et al. investigated the influence of a plant-based fiber-rich diet and fermented food on the intestinal microbiome and host-immune system in healthy individuals. As detected, a diet based on a high-fermented food diet elevated bacterial diversity while reducing inflammatory markers (18). Recent studies focusing on the diet-driven structure of bacterial communities in cancer patients outline the potential clinical benefits in the context of immunotherapy response modulation (14, 19, 20). Moreover, numerous data highlight the emerging impact of physical activity and exercise in combination with dietary interventions on the quality of life in cancer survivors (21–23).

Herein, we review current knowledge about the role of diet-related microbiome changes and physical activity in cancer patients. Specifically, we describe the host-microbiome interactions in various types of diets. In addition, ongoing clinical trials concerning the clinical implication of diet and exercise interventions in cancer patients are provided. According to the findings so far, targeting the gut microbiota through dietary habits in combination with regular physical activity might represent a potential trend in the future care of cancer patients. However, exact, clinically relevant correlations need to be evaluated in large clinical trials.

## 2 A diet-microbiome relationship in cancer patients

The impact of nutrition on the efficacy of anti-cancer therapy and patient outcomes is gaining more attention. Mueller et al. showed that pre-surgical administration of immunonutrition containing omega-3 fatty acids, arginine, RNA-nucleotides, and soluble guar fiber improved wound complications and decreased the length of hospital stay in patients with recurrent head and neck squamous cell carcinoma (24). A meta-analysis of ovarian cancer patients confirmed an inverse association between the total vegetable and fruit intake before diagnosis and overall mortality (25).

Different dietary patterns shape the gut microbiome composition and function (26–28). Clinical studies support the importance of a diet that would lead to a higher microbial diversity with limited inflammation-inducing microorganisms and molecules (18, 19, 29). The study on 115 colorectal cancer patients undergoing surgical intervention showed that a higher seaweed consumption correlated with a lower level of *Rikenellaceae* and *Alistipes* in the study cohort. On the other hand, data revealed an association between an increased proportion of Bacteroidetes, *Bacteroida*, and *Bacteroidales* and higher beverage consumption. The presence of butyrate-producing *Clostridium symbiosum* negatively correlates with fatty and amino acid intake (30).

### 2.1 High-fat diet

Ample data confirmed a correlation between high body mass index (BMI) and serious diseases, including cancer (31, 32). Obesity has been associated with a significant decrease in gut microbiome diversity (33). Fruge et al. found the impact of a diet on gut microbiome composition among 40 overweight and obese prostate cancer patients. Samples collected at baseline confirmed an elevated abundance of Proteobacteria. Increased poultry intake was associated with the prevalence of *Clostridiales*, while red meat consumption correlated with high *Prevotella* and *Blautia* (34).

A high-fat diet (HFD) induces microbial changes associated with dysbiosis (35). A meta-analysis of 27 dietary studies with 1,101 animal and human samples described significant microbiome alterations related to HFD, showing the most reproducible signals from the *Lactococcus* species (36). Currently, *in vivo* modulation of the gut microbiome by an HFD led to colorectal cancer development via gut microbiota dysbiosis, impaired metabolic pathways, and gut barrier disruption. The results showed an abundance of *Alistipes* and decreased *Parabacteroides distasonis* in HFD-fed animals (37). Matsushita et al. indicated the relationship between the elevated presence of systemic lipopolysaccharides related to HFD-induced microbiota disruption and the growth of inflammatory prostate cancer *in vivo* (38). Importantly, the Chinese traditional medicine extract Evodiamine helped to restore the gut microbiome, leading to a decreased amount of *Enterococcus faecalis* and *Escherichia coli* along with *Bifidobacterium* and *Lactobacillus* enrichment in the feces of HFD-fed mice with colorectal cancer (39).

### 2.2 Fiber-rich diet

A pooled analysis of 10 prospective cohorts with 1,445,850 million individuals documented a reduced risk of lung cancer after the consumption of dietary fiber with yogurt (40). In tumor-bearing mice, a high-fiber diet induced the IFN-1 production by intratumoral monocytes leading to a higher efficacy of immunotherapy (41). Clinical studies also confirmed positive correlations and improved response to immune checkpoint inhibitors in the group of patients with a high-fiber intake (42). High dietary fiber was associated with the consumption of fruit, vegetables, and calcium and improved progression-free survival (PFS) in 37 of 128 melanoma patients. A 5-gram increase in fiber intake correlated with a 30% decline in the risk of cancer progression or death. The results revealed the enrichment of the *Ruminococcaceae* family and *Faecalibacterium* genus in a group of patients supplemented with sufficient fiber. In an animal model, a diet rich in fiber led to a higher proportion of propionate in stool and correlated with delayed tumor growth (19). A lower consumption of fiber and omega-3 fatty acids was associated with poor response to immunotherapy in melanoma patients (43). Analysis of post-menopausal women with breast cancer confirmed that high total dietary fiber correlated with the decrease in *Clostridium* spp., showing a correlation between dietary fiber and bacterial taxons with β-glucuronidase activity (44). Fiber-rich diet increases the levels of microbiota-derived SFCA (45), while a low-fiber diet leads to bacterial utilization of amino acids and host mucins (46).

### 2.3 Mediterranean diet

Mediterranean diet (MD) is assumed to be involved in reduced cancer risk, but the mechanisms are still under discussion (47). A significant correlation between MD and a lower incidence of cancer was observed in the Greek (48), Afghanistan (49), and Italian populations (50). Accordingly, a meta-analysis of prospective cohort studies showed that MD reduced mortality from cancer or cardiovascular diseases (51). The European Prospective Investigation into Cancer and Nutrition (EPIC) study documented that vegetables and fruit protected against breast, colorectal, and lung cancer development. The consumption of alcohol, red, and processed meat correlated with colorectal cancer risk, while the MD and higher fish intake had a strong protective effect (52). However, the Netherland cohort study did not find an association between MD and reduced colorectal cancer risk in the Dutch population (53).

The positive impact of MD on inflammatory processes was observed in preclinical and clinical studies. MD improved azoxymethane-induced dysbiosis in the murine model of colorectal cancer on a high-fat Western diet. The prevalence of *Lactobacillaceae* was higher in the MD group of azoxymethane-treated animals following a low-fat diet (54). A combination of MD and a healthy lifestyle positively affected the quality of life in breast cancer survivors (55) and resulted in a reduction in overall mortality (56). The Bacteroidetes/Firmicutes ratio decreased in a group of breast cancer survivors supplemented with MD in combination with probiotic sachets containing *Bifidobacterium longum* BB536 and *Lactobacillus* rhamnosus HN001 (57). Another study reported reduced gastrointestinal complications in gynecologic cancer patients treated with platinum-based chemotherapy receiving MD (58). Moreover, a meta-analysis confirmed an association between the presence of anti-inflammatory microbiota and the consumption of MD in participants with colorectal cancer or gastrointestinal diseases (59).

### 2.4 Rice and beans diet

A higher intake of rice and beans was inversely associated with the risk of skin and esophageal cancer (60–62). Alterations in gut microbial composition leading to a higher diversity were identified in a randomized clinical trial evaluating the effects of heat-stabilized rice bran or cooked navy bean powder in colorectal cancer survivors. Both diet components similarly increased total dietary fiber. However, only rice bran intake decreased the Firmicutes/Bacteroidetes ratio and led to increased levels of fecal SCFA (63). Differences in a stool metabolome linked to gut microbial metabolism were observed between overweight and obese colorectal survivors supplemented with 35 g of cooked navy bean and navy bean-free snacks (64). Twenty eight-day diet program with heat-stabilized rice bran or navy bean powder in overweight or obese colorectal cancer survivors led to increased fiber intake. After 14 days, rice bran decreased Firmicutes/Bacteroidetes ratio and elevated fecal concentration of propionate and acetate. However, further longitudinal analyses are needed to evaluate the impact of heat-stabilized rice bran on cancer patient outcomes (63). Consumption of dietary rice bran for 24 weeks in participants with a high risk of colorectal cancer increased *Bifidobacteria, Prevotella_9*, and *Lactobacillales* in feces. However dietary intervention did not have a significant effect on fecal bacterial diversity (65). Zhang aimed to evaluate the impact of bean consumption on microbial composition in overweight or obese patients positive for precancerous polyps or colorectal cancer in a BE GONE study (66). The first results of this interventional study indicate that an eight-week boost in dry bean consumption potentially helps to enhance gut microbiome diversity in the study group (67).

### 2.5 Ketogenic diet

Numerous preclinical findings described the potential therapeutic impact of the ketogenic diet (KD) on tumor growth and enhanced immunity (68–70) and its positive correlation with the response to chemo- and radiotherapy (71, 72). KD, consisting of high fat, low carbohydrate, and low protein, causes metabolic changes via increased blood ketones and reduced blood glucose, leading to starvation of cancer cells. These metabolic changes suggest being implicated in improved survival of animals with malignant gliomas treated with chemotherapy (73). On the contrary, the results from the murine melanoma model did not confirm the association between KD and enhanced tumor growth (74). Shifts in microbial balance resulted in *Lactobacillus* and *Coriobacteriaceae* decline, while *Romboutsia* and *Akkermansia* enrichment in KD-fed mice bearing ovarian cancer (75). Ferrere et al. stated that KD led to an increase in *Akkermansia muciniphila, Ruthenibacterium lactatiformans*, and *Pseudoflavonifractor capillosus* together with a decrease in *Lactobacillaceae* in melanoma–tumor-bearing mice (76).

The effects of the KD and its impact on the gut microbiome need to be evaluated in clinical settings. In a study comprising breast cancer treated with chemotherapy, it did not bring benefits in terms of improved quality of life and physical activity (77). Recently, the Diet Restriction and Exercise-induced Adaptations in Metastatic Breast Cancer (DREAM) study investigates the impact of the calorie-restricted and KD with aerobic exercise during chemotherapy on tumor burden, quality of life, and treatment toxicity in patients with metastatic breast cancer (78).

### 2.6 Malnutrition and nutritional support

Investigating the role of the diet-microbiome relationship in malnutrition is in line with the findings that nutritional deficiencies significantly correlate with the response to anti-cancer therapy (79, 80). A high risk of malnutrition, observed in patients with head and neck cancer, is of high concern (81, 82), and early nutritional screening might help identify patients at risk (83). Nutritional support and prophylactic swallowing exercises, performed before dysphagia or problematic swallowing development, improved patient outcomes (84). Uncured malnutrition leads to reduced efficacy and tolerance of chemotherapy or radiotherapy, including elevated treatment toxicity, complications, and prolonged hospitality (85). According to the findings, malnutrition leads to death in almost 20% of cancer patients (86). Studies uncovered that whey protein isolates might be potential supplements that improve nutrition in cancer patients. Whey protein supplementation plus zinc and selenium led to increased levels of albumin and immunoglobulin G in 42 chemotherapy-treated cancer patients compared to the control group supplemented with maltodextrin oral snacks (87).

## 3 The role of physical activity and exercise in cancer patients

Growing evidence indicates that physical activity is safe for cancer patients undergoing cancer treatment having an impact on metabolic and inflammatory parameters (88, 89). However, intervention during and after treatment should be planned individually with modifications related to different cancer diagnoses (21, 90). Longer survival was observed in stage III colon cancer patients who were physically active and ate vegetables, fruits, and whole grains than participants with higher BMI who did not keep healthy behaviors (88). According to some findings, regular physical activity and exercise were shown to alter the gut microbiome independently from a diet (91). An analysis of ten cross-sectional and seven longitudinal studies documented an increased abundance of *Eubacterium rectale*, *Akkermansia muciniphila, Faecalibacterium prausnitzii, Eubacterium hallii*, and *Bifidobacterium* spp. in active individuals (92).

Beneficial outcomes of exercise-induced modifications in the murine gut microbiome and metabolome suggest being involved in the prevention and treatment of intestinal inflammation and cancer (93). The effect of diet and exercise in breast cancer patients, stage 0-II through a presurgical weight-loss plan (nutritionally adequate and energy-restricted diet with 30 min exercise/day) led to a higher bacterial richness and diversity. According to the level of *Akkermansia muciniphila*, the women were divided into the high and low *Akkermansia muciniphila* groups (HAM and LAM group, respectively). The analysis of stool samples from the HAM group showed an increased *Prevotella* and *Lactobacillus* and reduced *Clostridium*, *Campylobacter*, and *Helicobacter*. During the study course, women belonging to the HAM group lost a significant percentage of body fat, while the obtained results were not significant for the LAM group (94). Interestingly, the results of the study by Newton et al. will evaluate the effect of a 3-month exercise program on the expansion of favorable gut bacteria in men with prostate cancer receiving androgen deprivation therapy (95).

Paulsen et al. found an association between gut microbiota beta diversity, fatigue, depression, cardio-respiratory fitness, and exercise in breast cancer survivors (96). The term prehabilitation means increased physical fitness intending to reduce stress before esophagogastric surgery (97). The results of a randomized clinical trial showed that prehabilitation increased physical status, endurance, and walking in patients undergoing a surgical procedure for malignant gastroesophageal cancer (98). Uster et al. confirmed that a combination of nutritional sessions and physical exercise in patients with metastatic tumors of the GIT or lung tract contributed to adequate protein intake. As shown, nausea and vomiting were less presented in study participants. Individual nutritional plans for patients contained measures such as oral nutritional supplements, snacks rich in proteins, and snacks rich in energy. Medical history such as anti-cancer therapy, drugs, and blood parameters was documented by the dietician. The exercise program lasted 60 min twice a week with the participation of a physiotherapist (99).

Traditional MD, physical activity, and vitamin D supplementation improved health-related quality of life in breast cancer survivors due to reduced body weight and relieving symptoms of treatment-associated toxicity (55). The result of the survey 12 months after the adaptation of the lifestyle modification program, including higher dietary fiber, lower saturated fatty acids intake, increased exercise, and elevated levels of circulating vitamins, showed a decreased occurrence of symptoms such as nausea, fatigue, vomiting, and constipation in patients with healthy regimen (55). Ho et al. evaluated the effect of diet and physical activity on generic and cancer-specific quality of life, anxiety, and depression in a cohort of 223 patients with colorectal malignancies. According to the findings, the dietary intervention improved both quality of life and depression levels. However, physical activity did not lead to significant improvements (100).

The impact of diet- and exercise-driven changes on microbial diversity in cancer patients is challenging (Table 1) and depends on numerous factors, including cancer type, the stage of disease, specific training interventions, and the patient’s health status.

**Table 1:** Beneficial effects of dietary and exercise interventions in cancer patients.

Study	Study design	Disease	Purpose	Patients (n)	Intervention	Study status
Microbiome and dietary interventions						
NCT04869956	An interventional controlled randomized open-label study parallel assignment	Colorectal cancer	To evaluate the changes in the gut microbiome and determine serum levels of inflammation markers in blood samples	50 adults	Patients will follow a diet containing standard nutritional recommendations or a high-fiber diet with 30 grams of fiber/day at least 1 month before the surgical procedure.	Recruiting
NCT04753359	An interventional randomized study parallel assignment	Colorectal cancer	To study fecal levels of bile acids and assess the metabolic function of gut microbiota	232 adults	Participants will receive a weight-stable MD or daily calorie restriction (−500–750 kcal/day).	Recruiting
NCT04079270	An interventional randomized study parallel assignment	Breast cancer	To analyze the efficacy of a personalized diet vs. a standard low-fat MD to control body mass and investigate potential changes in the gut microbiota	200 adults	Participants will be randomly assigned to either follow a low-fat diet or receive personalized dietary guidance for a duration of 6 months. Smartphone applications will help to monitor their daily food consumption and physical activity.	Recruiting
NCT05387876	An interventional double-blind placebo-controlled randomized study parallel assignment	Colorectal cancer	To confirm whether vitamin D might be responsible for changes in the microbiome structure and its function	43 adults	Vitamin D gummies or organic gummy candies without vitamin D (placebo) will be administered to patients once per day for a 3-month period.	Active, not recruiting
NCT05643859	An interventional open-label study single-group assignment	Benign colorectal neoplasm/non-neoplastic anal disorder	To evaluate the effect of fiber supplementation on alternations in taxa abundance and microbiome diversity	100 adults	Participants undergoing proctoscopy or anoscopy will receive fiber through oral administration.	Recruiting
NCT02843425	An interventional randomized open-label study crossover assignment	Prevention of colorectal cancer	To characterize whether canned, pre-cooked beans enhance favorable gut bacteria and mitigate the effect of obesity on cancer risk	71 adults	For the first 2 weeks, patients with precancerous colon or rectum polyps and colorectal cancer survivors will add 1/2 cup of canned beans per day to their dietary regimens.	Active, not recruiting
NCT03550885	An interventional randomized study crossover assignment	Colorectal cancer	To identify changes in mucosal abundance of bacterial genes associated with sulfur and bile acid metabolism and observe altered serum bile acids	44 adults	Patients will receive a 3-week controlled isocaloric Western-type diet (high taurine and saturated fat diet) or an isocaloric diet predominantly composed of plant-based food.	Active, not recruiting
NCT05516641	An interventional randomized study parallel assignment	Rectal cancer	To explore changes in gut microbiome and evaluate the impact of prebiotics on the tumor immune profile	20 adults	Soluble corn fiber or maltodextrin (placebo) will be added once daily to the diet during patient neoadjuvant treatment.	Recruiting
NCT05195970	An interventional open-label study single-group assignment	Colorectal cancer	To study the composition of gut microbiome, taxonomy changes, bacterial diversity changes, and urolithin levels in urine	200 adults	During the study, individuals will incorporate a daily addition of 2 ounces of walnuts into their regular diet for a duration of 29 days as part of the dietary intervention.	Recruiting
NCT04645680	An interventional randomized study parallel assignment	Metastatic melanoma	To investigate the impact of two different diets on microbiome changes, metabolic profile, quality of life, and the incidence of adverse events	42 adults	Over the course of 11 weeks, individuals undergoing conventional immunotherapy (using pembrolizumab or nivolumab) will follow a nutrition plan consisting of isocaloric whole foods higher in fiber vs. standard whole foods.	Recruiting
NCT04940468	An interventional open-label study parallel assignment	Leukemia/lymphoma	To characterize whether dietary intervention might reduce *Clostridium difficile* infection by quantification of toxins in stool and analyze the gut microbiome	124 children/ adults	Patients experiencing their initial or second episode of *Clostridium difficile* infection will follow a high-fiber diet or no dietary modifications.	Recruiting
NCT05061316	An interventional open-label study single-group assignment	Head and neck cancer	To define the effect of pre-operative nutritional intervention (immunonutrition drink) on the gut microbiome and characterize the gut microbiome associated with decreased post-operative complications	30 adults	In malnourished patients, Nestlé Impact Advanced Recovery drink supporting the immune system will be administered orally or through a feeding tube two times daily for 5 days before the surgery.	Recruiting
NCT04985565	An interventional open-label study single-group assignment	Prostate carcinoma	To investigate tolerance of MD and its effect on fecal microbiome and metabolic parameters	10 adults	MD will be administered 6 days per week for 4 weeks before standard radical prostatectomy.	Recruiting
NCT05296681	An interventional open-label study parallel assignment	Metastatic colon carcinoma	To characterize the role of intervention related to restoration of gut microbiome, improved gut barrier function, and assess the occurrence of treatment-related adverse events	42 adults	NBT-NM108, rich in dietary fiber, will be consumed 4 times a day before meals and 2 h after dinner for a period of 56 days. The supplementation will start 5 days prior to chemotherapy.	Recruiting
NCT05471414	An interventional randomized open-label study parallel assignment	Prostate cancer	To analyze the changes in the diversity of the gut microbiome from baseline and determine serum levels of adiponectin, leptin, glucose, and inflammation markers	60 adults	Patients will eat a home-delivered whole-food, plant-based diet prepared by Plantable for a duration of 8 weeks, followed by diet coaching via phone calls, SMS, e-mails, and mobile applications in the subsequent 18 months. The second group of patients will receive only weekly nutritional counseling for 18 weeks.	Recruiting
NCT04965129	An interventional randomized study parallel assignment	Lung cancer	To assess the improvement in muscle mass, therapeutic response, and modulation of the gut microbiome within a 4-month period	50 adults	Patients treated with immunotherapy, chemotherapy, and tyrosine kinase inhibitors will receive a high protein diet plus supplementation with fish oil vs. placebo olive oil pill manufactured to mimic fish oil.	Recruiting
NCT03087903	An interventional open-label study single-group assignment	Prostate cancer	To define proinflammatory cytokines, lipid and metabolic panel in serum samples and study the gut microbiome and stool metabolomics	20 adults	150 mg of grape seed extract product as Leucoselect Phytosome preparation will be administered twice daily for 1 year.	Recruiting
NCT05135351	An interventional randomized study parallel assignment	Multiple myeloma/lymphoma	To analyze the changes in gut microbiome diversity between placebo and resistant starch arms and study differences in the gut permeability during transplant	30 adults	A prebiotic nutritional supplement (resistant potato starch) vs. maltodextrin as a placebo will be administered to patients with hematologic malignancies undergoing auto-HSCT.	Recruiting
NCT04231734	An interventional open-label study single-group assignment	Mantle cell lymphoma	To identify the effect of a KD on gut microbiome and changes in body composition	8 adults	Patients will eat 3 ketogenic meals per day for up to 12 weeks.	Recruiting
NCT04821258	An interventional randomized study parallel assignment	Colorectal cancer	To rate the effect of supplements on the complications, adverse events, and gut microbiome composition after surgical intervention	144 adults	Participants will follow MICODIGEST 2.0 supplementation containing nine different fungal extracts (*Ganoderma lucidum*, *Agaricus blazei*, *Grifola frondosa*, *Hericium erinaceus*, *Cordyceps sinensis*, *Inonotus obliquus*, *Pleurotus ostreatus*, *Polyporus umbellatus*, and *Lentinula edodes*) or placebo.	Unknown
Microbiome and exercise programs						
NCT03314688	An interventional randomized study parallel assignment	Breast neoplasms	To assess the impact of a 1-year intervention following dietary and physical activity guidelines vs. usual care on the gut microbiome, body mass index, and body parameters	173 adults	Participants will receive usual care vs. motivational counseling to follow established dietary and exercise recommendations with scheduled neoadjuvant or adjuvant chemotherapy.	Active, not recruiting
NCT05539794	An interventional randomized study parallel assignment	Adolescent cancer	To study the multisystemic benefit of exercise on improved immune function, metabolic and inflammatory markers, and changes in the gut microbiome (alpha-diversity, beta-diversity, specific bacteria) from baseline to the end of treatment	136 children	The exercise program consists of 3 supervised sessions/week of aerobic and muscle-strengthening exercises along with specific respiratory muscle training for 5 days/week. The counseling component includes bi-monthly nutritional support and monthly educational sessions for family members.	Recruiting
NCT04866810	An interventional randomized open-label study parallel assignment	Melanoma	To determine whether nutritional intake and exercise routines can influence gut microbiome composition	80 adults	Patients receiving immunotherapy will eat a plant-based, high-fiber diet. The exercise plan will be scheduled for 150 min of moderate or 75 min of high-intensity exercise per week.	Recruiting
NCT05000502	An interventional randomized study parallel assignment	Breast cancer	To investigate whether exercise affects microbial diversity and taxa in fecal samples	40 adults	Patients will perform a home-based program involving weekly video conferences with exercise specialists for 10 weeks. A systematic increase in duration and intensity will enhance cardiorespiratory fitness.	Recruiting
NCT04088708	An interventional randomized study parallel assignment	Breast cancer	To evaluate the impact of exercise on the number, distribution, and types of bacteria in the gut of patients and determine serum levels of inflammation markers	126 adults	Exercise specialists will prepare progressive aerobic training sessions lasting 20–60 min. The intensity of exercises will gradually increase.	Recruiting
NCT05686213	An interventional randomized open-label study parallel assignment	Cancer	To assess participation rate, exercise intervention attendance, and rate body composition, muscle strength, physical activity, and health-related quality of life together with the shifts in microbial communities	39 adults	One group of patients will be enrolled in a supervised exercise program with aerobic and resistance exercises, held twice weekly for 60 min. The other group will perform aerobic exercise 5 days a week for 30 min before their daily radiotherapy sessions.	Recruiting
NCT05238376	An interventional non-randomized open-label study single-group assignment	Hematologic malignancies	To document changes in physical function/activity and mental health and analyze oral and stool microbial diversity	70 adults	90 days after allo-HSCT, patients will follow a fitness regimen comprising 12 weeks of high-intensity interval training (3 times a week), and resistance training (2 times a week).	Recruiting
NCT04706676	An interventional randomized open-label study parallel assignment	Cancer	To monitor markers of metabolic syndrome, body composition, hospitalized days, and analyze the gut microbiome	127 children	Integrative neuromuscular training consists of appropriate activities for training strength, power, motor skill training, dynamic stability, core-focused strength, plyometric, and agility.	Recruiting
NCT05312255	An interventional non-randomized open-label study parallel assignment	Plasma cell myeloma	To compare the fecal microbiome between the group with physical exercise vs. the group with intermittent fasting	150 adults	Strength training sessions, performed twice weekly for 6 months, aim to increase physical activity. Another group of patients will undergo 1-month intermittent fasting.	Recruiting

The table summarizes ongoing clinical trials focused on the impact of diet and supervised training sessions on gut microbiome diversity and the quality of life among patients (according to https://ClinicalTrials.gov/, processed July 28th, 2023).allo-HSCT; allogeneic hematopoietic stem cell transplantation; KD; ketogenic diet, MD; Mediterranean diet.

## 4 Conclusion and future directions

Targeting the gut microbiome in cancer patients represents an emerging approach that is clinically relevant, not only in terms of patient care but also as a preventive tool, mainly in gastrointestinal cancers. Mounting evidence emphasizes the potential of gut microbiota modulations in improving cancer treatment efficacy, mainly chemo- and immunotherapy. However, large clinical trials are needed to better elucidate the relationship between diet- and exercise-related microbiota modulations and cancer patient outcomes. Precise determination of treatment response-favorable bacterial taxons and communities could bring microbiota-based interventions into clinical practice. Importantly, studies evaluating the correlations between various types of diet, and the particular composition of the gut microbiome, may bring clinically relevant results. In this context, clinicians should collect as much information about the patient‘s nutritional status and eating habits as possible, even in the pre-treatment period.

Standardization of methods and outcome measures are necessary to address the safety and efficacy issues. Diet and fitness plans for patients should be created based on consultations with specialists. Multidisciplinary cooperation between clinicians, nutritionists, and physical therapists might help to increase patients’ response to anti-cancer treatment and improve quality of life while reducing treatment-associated side effects. Educational programs concerning the benefits of a healthy and high-quality diet might be helpful for patients’ engagement.

Moreover, it is crucial to design longitudinal trials that use not only surrogate endpoints for the determination of the effect of nutrition on microbiota but include clinically relevant endpoints as well. Multiple factors influencing microbial composition need to be taken into account when evaluating interactions between diet and microbiota. Especially in cancer patients with progressing disease, we need to distinguish the association from the causal relationship between changes in diet/physical activity and microbiota changes. On the other side, emerging evidence suggests how microbiota modulates treatment response to anti-cancer drugs, and this is most prominent in the era of new-generation immunotherapies. Therefore, strategies to maintain and/or positively modulate gut microbiota could improve cancer care outcomes.

## Author contributions

SC: Conceptualization, Funding acquisition, Project administration, Visualization, Writing – original draft, Writing – review & editing. AS: Visualization, Writing – original draft. VS: Visualization, Writing – review & editing. MM: Supervision, Writing – review & editing.
